# Cholecystokinin and the hormone concept

**DOI:** 10.1530/EC-21-0025

**Published:** 2021-02-25

**Authors:** Jens F Rehfeld

**Affiliations:** 1 Department of Clinical Biochemistry, Rigshospitalet, University of Copenhagen, Copenhagen, Denmark

**Keywords:** cholecystokinin, bioactive peptides, hormone concept, growth factors, neurotransmitter peptides

## Abstract

The birth certificate for endocrinology was Bayliss’ and Starling’s demonstration in 1902 that regulation of bodily functions is not only neuronal but also due to blood-borne messengers. Starling named these messengers hormones. Since then transport via blood has defined hormones. This definition, however, may be too narrow. Thus, today we know that several peptide hormones are not only produced and released to blood from endocrine cells but also released from neurons, myocytes, immune cells, endothelial cells, spermatogenic cells, fat cells, etc. And they are often secreted in cell-specific molecular forms with more or less different spectra of activity. The present review depicts this development with the story about cholecystokinin which was discovered in 1928 as a hormone and still in 1976 was conceived as a single blood-borne peptide. Today’s multifaceted picture of cholecystokinin suggests that time may be ripe for expansion of the hormone concept to all messenger molecules, which activate their target cells – irrespective of their road to the target (endocrine, neurocrine, neuronal, paracrine, autocrine, etc.) and irrespective of their kind of activity as classical hormone, growth factor, neurotransmitter, adipokine, cytokine, myokine, or fertility factor.

## Introduction

The word hormone originates from the Greek word ’hormoa’. It was proposed by the British linguist WB Hardy and introduced by Ernest Starling in his Croonian Lectures published in ‘The Lancet’ in 1905 (1) – 3 years after his and William Bayliss’ breakthrough discovery of the first hormone in history, secretin (2). ‘Hormone’ was rapidly accepted as a general designation for blood-borne chemical messengers of which secretin was the first and gastrin the second example (2, 3). Accordingly, hormones and blood-borne regulation became core-concepts in endocrinology as complementary to neuronal regulation, which until then had been considered the only way for regulation and coordination of bodily functions (4, 5).

In the wake of the secretin discovery, endocrinology blossomed with uncoverings of a multitude of additional hormones, endocrine glands and ensuing paradigmatic shifts in the understanding of regulatory physiology. Chemically, the structure of hormones turned out to vary from proteins and peptides, to steroids, thyronins and monoamines. And cellularly, most new hormones appeared to originate from glands such as the pituitary, thyroid, parathyroids, pancreatic islets, adrenals, ovaries and testes. A major exception from the glandular origin, however, was the gastrointestinal hormones, because endocrine cells in the gut are distributed in a regional manner among non-endocrine cells in the gastrointestinal mucosa and not collected in the glands. Hence, we have the puzzling paradox that many endocrinologists do not consider the gut to be a classic endocrine organ, although the gastrointestinal tract by all parameters is the largest and in evolutionary as well as historical terms the oldest endocrine organ in the body (for reviews, see 6, 7, 8, 9).

For hormones in general, however, the progress in cellular and molecular biology during the last decades has in a fundamental manner deepened the insight into a new biology (10). This insight has changed both basic and clinical endocrinology. The following story about cholecystokinin (CCK) illustrates how studies of a single peptide hormone from the gut (CCK-33) has gradually contributed to expand endocrinology into a considerably wider concept than just being about blood-borne messenger molecules.

## The cholecystokinin (CCK) story

### Six chronological descriptions of CCK as a blood-borne hormone

#### The prehistory

Almost a century before CCK was discovered in 1928 as a gallbladder-emptying hormone (11), European physiologists took a broad interest in bile secretion and the role of bile in digestion (for reviews, see 12, 13). For instance, Claude Bernard (who introduced the concept of ‘sécrétion interne’) reported in 1856 that installation of hydrochloric acid into the duodenum increased the secretion of bile (14). In 1903, another French physiologist, Wertheimer, reported that duodenal stimulation of bile secretion persisted after cutting vagal and sympathetic neurons to the duodenum (15). And in the same year, Fleig described how blood from an isolated loop of the small intestine, into which acid was injected, increased bile-flow when transfused into another dog (16). Thus, already 1 year after Bayliss’ and Starling’s discovery of secretin, French physiologists had evidence to suggest that the duodenum might release a blood-borne chemical messenger or hormone that stimulated bile secretion. But they were not sure whether the bile-stimulating effect was still to some extent caused by secretin. During the following years, Okada studied bile secretion in Starling’s laboratory in London. And in 1914, he reported that acid in the duodenum not only stimulated the secretion of hepatic bile but it also emptied gallbladder bile into the small intestine (17).

In retrospect, it may be surprising that so many bile secretion studies over almost a century did not ignite the idea that the upper small intestine harbored a specific bile-releasing hormone, different from secretin. Therefore, further experiments were necessary in order to rule out a possible bile-secretagogue activity of secretin. Such studies were eventually performed by Andrew Ivy and colleagues in Chicago in the late 1920s (11, 18, 19, 20).

#### The functional identification

Ivy *et al.* examined first whether different preparations of secretin of various purity affected the gallbladder in dogs (20). They concluded – pretty inconclusively – from this study that an observed gallbladder contracting activity was due to ‘secretin or some substance closely associated with it’ (13, 20). After further cross-circulation studies in dogs, they saw that hydrochloric acid in the duodenum of a dog caused gallbladder contraction in another. And when they kept that observation together with differences in solubility of secretin preparations and preparations containing the gallbladder contracting activity, they concluded that the small intestine produced a new hormonal activity which they decided to name cholecystokinin (11, 18, 19, 20). Thus, a third and separate gut hormone-like activity was entering the scene. And from being concerned mainly with secretin and a little with gastrin, gastrointestinal endocrinology broadened. Today, secretin, gastrin, and CCK are – for good reasons – accepted as the classical troika of gut hormones. Nevertheless, compared to secretin, the interest in CCK was limited in the following decades. In 1946, Ivy therefore tried to evoke clinical interest for CCK by suggesting that CCK injections might be of use for the diagnosis and therapy of biliary dyskinesia (21). But the response from clinicians remained meager.

In the meantime, another hormonal activity from the duodenal mucosa had been found by Harper and Raper in 1943 (22). Its existence was rapidly confirmed in Ivy’s laboratory (23). The active substance was named pancreozymin, because it stimulated the secretion of pancreatic enzymes. Pancreozymin was for more than 20 years considered a separate gut hormone (22, 23, 24). It was noted, however, that pancreozymin preparations also stimulated gallbladder contraction (25). This side effect was, however, considered to be due to contamination of the partly purified pancreozymin preparations with CCK. The situation illustrated the need for pure hormone preparations that hopefully also would allow identification of the structure of the hormones.

Already from 1912, attempts had been made in several laboratories to purify and isolate secretin from intestinal extracts (26, 27, 28, 29). The goal was – as just mentioned – to have a pure and stable secretin preparation for physiological and clinical tests of exocrine pancreatic functions (30). Similarly, attempts to purify CCK was also initiated early (19, 31). In retrospect, however, these purification attempts were premature and too optimistic because the necessary biochemical technology was not yet available (for review, see 32). Peptide purification required at least electrophoretic, ion-exchange chromatographic and counter current distribution techniques, which became available in the 1950s.

#### The structural identifications

The identification of the CCK structure was again associated with secretin. After pilot extractions and early purification steps in the late 1940s and early 1950s (30, 31), Jorpes and Mutt in Stockholm decided to establish a large-scale platform of almost industrial size for extractions of porcine small intestines in order to have sufficient material for purification of secretin, CCK and pancreozymin (33, 34). That was a wise decision, because the intestinal tissue concentrations of both secretin and CCK have turned out to be low (35, 36). Mutt and Jorpes managed to collect the proximal 1 m small intestine from 20,000 hogs (20 km of intestine!). After boiling, acetic acid extractions, absorption to alginic acid, fractionations with ethanol and methanol, ion-exchange and later size chromatographies, and finally counter current distributions, they had approximately 10 mg essentially pure peptides for sequence analysis (37, 38, 39).

**Figure 1:**
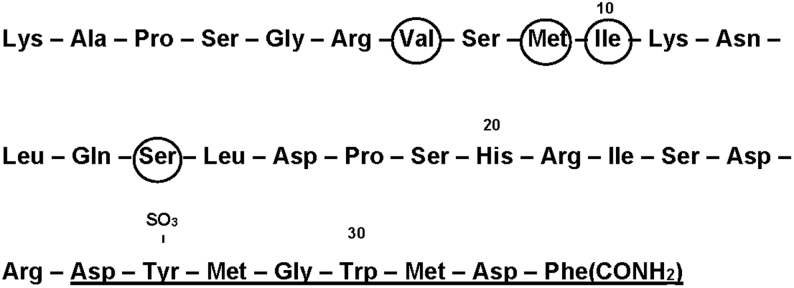
The amino sequence of porcine cholecystokinin-33 (CCK-33), the originally identified CCK-peptide (Mutt & Jorpes (39)). The bioactive CCK-8 sequence (26, 27, 28, 29, 30, 31, 32, 33) is underlined. Only the amino acids in the encircled positions no. 7, 9, 10, and 15 differ from those in the human sequence (Met, Ile, Val, and Asn, respectively).

**Figure 2:**
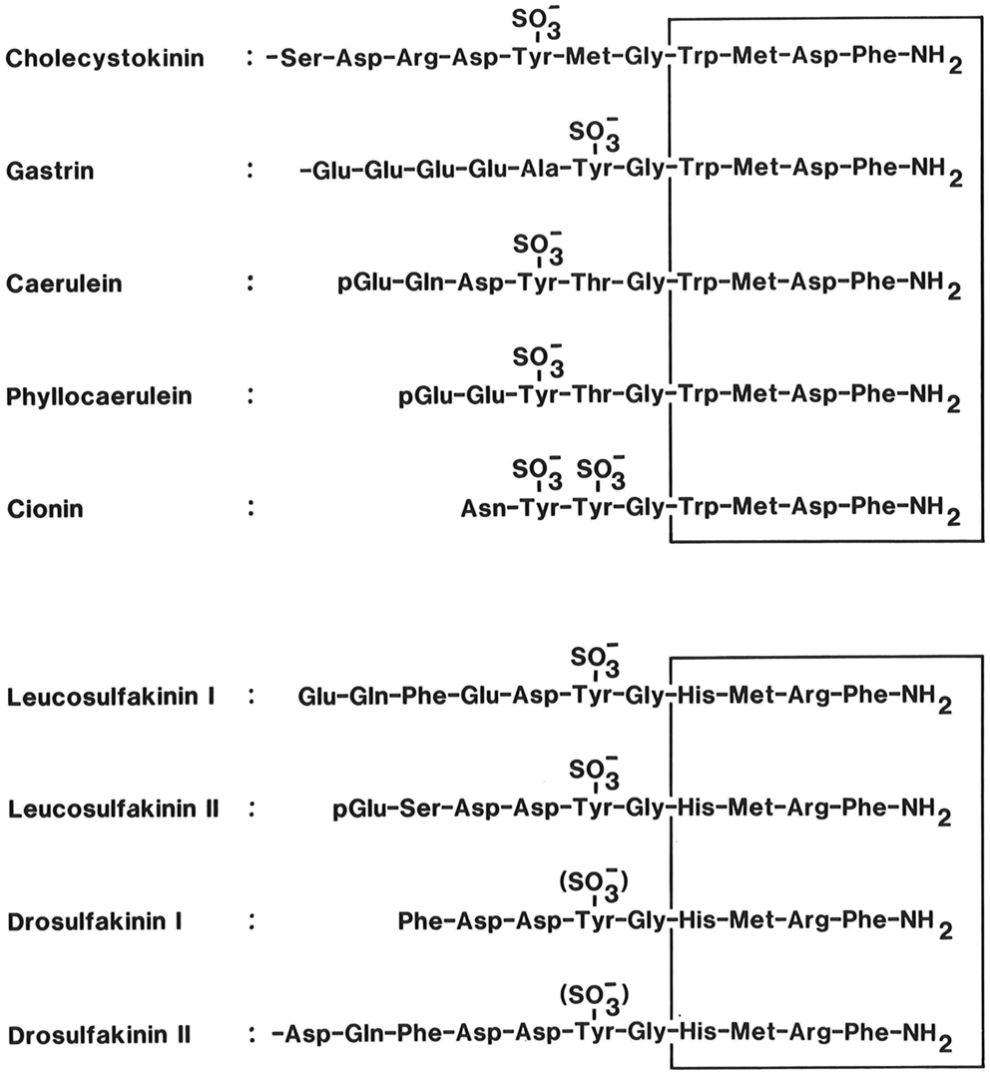
The bioactive sequences of peptide systems belonging to the cholecystokinin (CCK) family. CCK and the antral hormone, gastrin (43, 44), are the only mammalian members of the family. Caerulein and phyllocaerulein are identified from frog skin extracts (45, 46). Cionin is a neuropeptide isolated from the central ganglion of the protochord, ciona intestinalis (47). Note the unique disulfated sequence, which might suggest that cionin may resemble a common ancestor of CCK and gastrin (48). The core of the bioactive sequences, the common C-terminal tetrapeptide amide, is boxed. The lower panel shows the bioactive sequences of the insect neuropeptides, the sulfakinins, which are homologous to vertebrate and protochordian members of the CCK family (49, 50). Also their C-terminal tetrapeptide amide sequence is boxed.

This more than 20 years’ tour de force is probably the most important single effort in furthering gastrointestinal endocrinology. And the results were worth the effort: After identification of the secretin structure, CCK was identified as a tyrosyl-sulfated and carboxyamidated peptide of 33 amino acids, now called CCK-33 (Fig. 1). But the bioassay monitoring of gallbladder contractions and pancreatic bicarbonate and enzyme secretion of the different purification steps also revealed that CCK and pancreozymin was one and the same peptide hormone (40). After some discussion, consensus was obtained about maintaining the name cholecystokinin and its acronym, CCK, rather than the ungainly double name cholecystokinin-pancreozymin, or CCK-PZ (41, 42). Moreover, the structure also showed an unexpected close homology between the bioactive C-terminal sequences of CCK and that of the in 1964 sequenced gastric hormone, gastrin (43, 44) (Fig. 2).

The homology story, however, turned out to be even greater. In addition to the mammalian gastrins, the CCK-structure showed an even higher degree of homology with the simultaneously isolated frog skin peptides, caerulein and phyllocaerulein (45, 46). And particularly interesting in evolutionary terms was the disulfated cionin-peptide from the central ganglion of the protochordate, Ciona Intestinalis, which revealed a hybrid structure (47) similar to that of the presumed common ancestor of CCK and gastrin (48). Finally, also insects express a group of neuropeptides, the sulfokinins, with significant homologies to the CCK structure (49, 50) (see also Fig. 2). Thus, CCK represents a large family of potent bioactive peptides in the animal kingdom that in phylogenetic terms is 500–600 million years old (51, 52, 53).

Knowledge of the CCK-33 structure was a decisive milestone. Viktor Mutt generously supplied purified CCK-33 from Stockholm to interested research laboratories for functional studies, antibody production and chromatographic calibrations. And with the almost coincident chemical synthesis of the bioactive C-terminal octapeptide sequence (CCK-8), material became available for further CCK-research (54). Subsequent chromatographic studies on intestinal extracts showed, however, that CCK-33 was only one among several bioactive CCK-peptides (36, 55, 56, 57, 58). These other CCKs have been purified and their structures determined to be CCK-58, CCK-22, CCK-8 and CCK-5. The longer forms are released in both tyrosyl-sulfated and non-sulfated forms (59), whereas CCK-5 only exists as non-sulfated in lack of a tyrosyl residue for O-sulfation (58, 60). Thus, CCK is in molecular terms a highly heterogenous peptide system. Understanding of this molecular heterogeneity requires, however, insight into the CCK-gene expression cascade with emphasis on the posttranslational maturation of proCCK-expressing cells.

#### The CCK biogenesis

**Figure 3:**
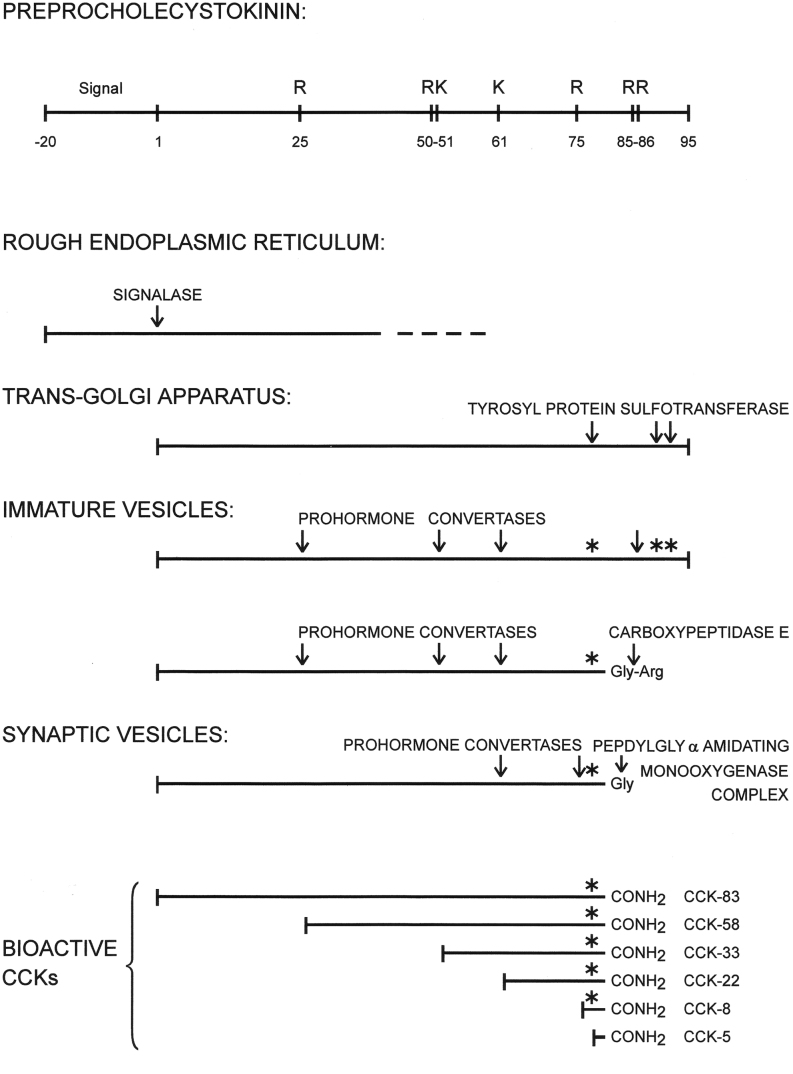
The posttranslational maturation of preprocholecystokinin in the endocrine I-cells of the small intestine. Mono-and dibasic cleavage sites (arginyl (R) and lysyl (K) residues) are indicated on the schematic figure of preprocholecystokinin (upper part of the figure). In the remaining part of the figure, the asterisks (*) indicate tyrosyl O-sulfation sites on the procholecystokinin processing intermediates and bioactive endproducts. Decisive processing enzymes (sulfortransferases, prohormone convertases, carboxypeptidase E, and the amidation enzyme (pepdityl-glycyl-α-amidating monooxygenase complex (PAM)) are also mentioned at the cellular level of the posttranslational processing pathway, where they act. In the intestinal endocrine I-cells, prohormone convertase 1/3 (PC 1/3) is predominant, whereas prohormone convertase 2 (PC 2) predominates in cerebral CCK neurons (see also 62). CCK-83 has been identified in tissue extracts but not in plasma (63).

The CCK gene was cloned and sequenced from a rat cell line in 1985 by Deschenes *et al.* (61). Only two of its three exons are coding. They are transcribed to one mRNA of 750 bases of which 345 are translated to a preproCCK protein of 115 amino acids (Fig. 3). The first part of the preproprotein is the signal peptide that is cleaved off by a signalase, leaving intact proCCK. Then follows a spacer-peptide in whose sequence there are several variations from species to species (51). The spacer sequence is followed by the CCK-58 sequence with only few species variations (51). The intestinal CCK-58 sequence undergoes extensive endoproteolysis by primarily prohormone convertase 1/3 at monobasic and a single dibasic cleavage site (62). As a result, the endocrine I-cells in the intestinal mucosa contain in their secretory granules a mixture of CCK-58, -33, -22, -8 and -5 of which CCK-33 appears to be the predominant form in the human intestine and circulation (63). As mentioned previously, however, 20–30% of the bioactive CCK-peptides in the small intestine are not tyrosyl-sulfated (59). The non-sulfated, but still carboxyamidated, CCK-peptides are not agonists for the CCK_1_-receptor and are consequently without effect on hepatic bile secretion and emptying of the gallbladder. But they are still bioactive as agonists for the CCK_2_ receptor. They function – in other words – as intestinal gastrins (64).

As indicated by Fig. 3, the cellular posttranslational maturation of proCCK involves multiple often incomplete processing steps catalyzed by enzymes that have been reviewed in detail elsewhere (62, 65, 66, 67). After release of the CCK peptides from enteroendocrine I-cells to blood the plasma pattern changes, because the longer molecular forms (CCK-58, -33 and -22) are cleared at a slower rate from circulation than the short CCKs (CCK-8 and -5).

#### The hormonal functions and receptors

The CCK-synthesizing I-cells in the gut mucosa have an apical membrane in direct contact with the intestinal lumen where it can taste the luminal content. The basal cell region is close to capillaries, to which the CCK-containing secretory granules are released upon stimulation (68, 69). The most important stimulus is food, in particular protein- and fat-rich food (70, 71). Of the major constituents, protein, L-amino acids and digested fat cause the largest release of CCK-peptides (70, 72). The chain length of the fatty acids determines the magnitude of the CCK response to lipids with long-chain fatty acids being more stimulatory than medium and short chain (73, 74, 75, 76). The response to carbohydrates is lower but still significant (77, 78).

The I-cells in the intestinal mucosa have the highest density in the duodenum and the proximal jejunum (59, 68, 69, 79). But there are still fair amounts of I-cells and CCK in the remaining small intestine and even a few in the colon (59, 79, 80). It is a mistake to consider the duodenum as the main source of intestinal CCK. Careful quantitation shows that the jejunum contains considerably more CCK, and even the ileal mucosa also expresses more bioactive CCK peptides than the duodenum (59). The explanation is simple: The duodenum constitutes only a short part of the small intestine. Besides, some immunohistochemical countings of I-cells in the duodenum have been false high due to use of antibodies that cross-react with duodenal gastrin cells (G-cells) that can be quite abundant (59, 69).

**Figure 4:**
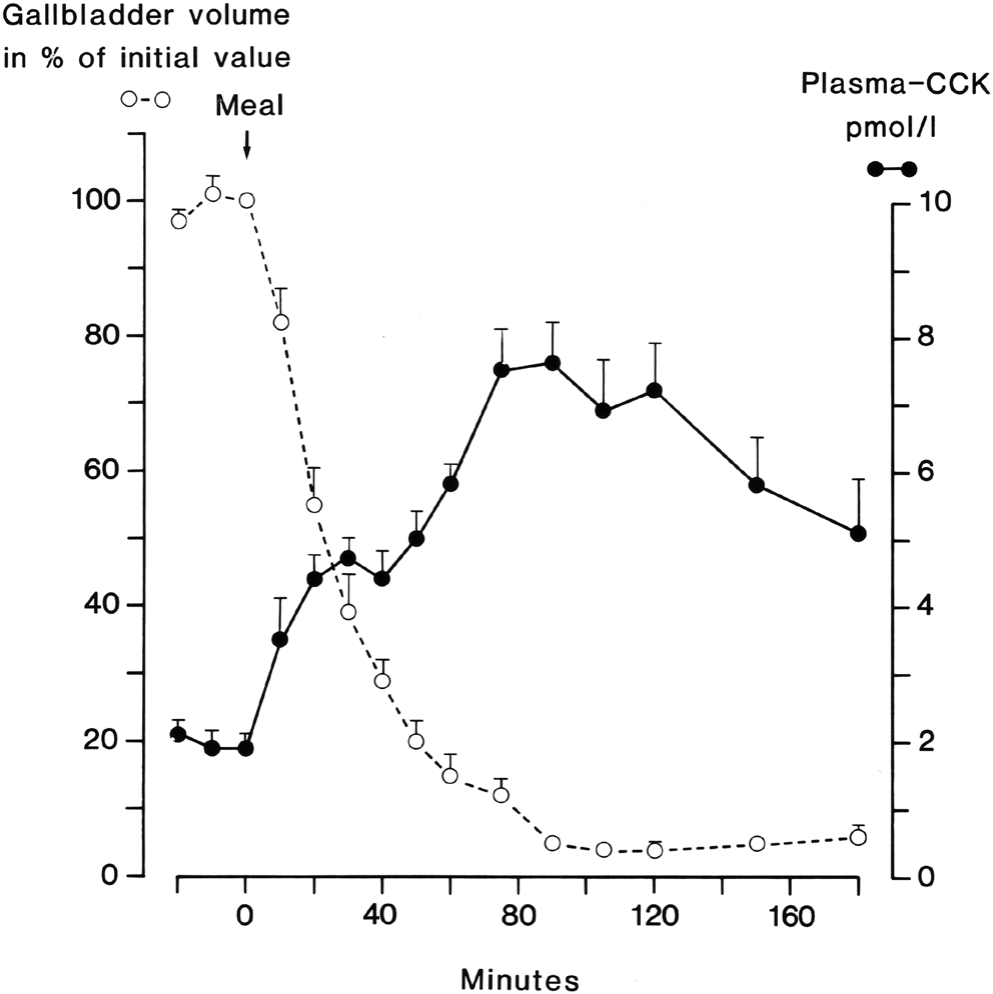
The concentrations of bioactive CCK in plasma vs gallbladder emptying during a mixed meal in normal human subjects. Data from (141) with permission.

Bioactive CCK peptides in blood circulate in the low picomolar range (63, 70, 71, 72). They potently stimulate their target cells via one of the two CCK receptors (81, 82) expressed on the target cell membrane. The CCK_1_ and CCK_2_ receptors are both of the GPRC type and show extensive homology (81, 82, 83). The CCK_1_ receptor, earlier named the CCK-A receptor, is also called the alimentary receptor. It mediates hepatic bile secretion, gallbladder contraction (Fig. 4), relaxation of the sphincter Oddi, pancreatic enzyme secretion and growth, inhibition of gastric acid secretion via somatostatin cells and inhibition of gastric emptying (84), satiety via afferent vagal fibers (85), and gut motility. Only tyrosyl-sulfated CCK peptides are agonists for the CCK_1_ receptor, which do not bind non-sulfated CCK peptides, nor any gastrins (81).

The CCK_2_ receptor is primarily expressed in the brain – and therefore earlier named the brain or CCK-B receptor. The CCK_2_ receptor was, however, originally cloned and structurally identified as the gastrin receptor (82). Accordingly, it is also expressed in the stomach with particularly high density on enterochromaffin-like (ECL) cells and occasionally on parietal cells. The CCK_2_ receptor therefore mediates gastrin’ergic acid secretion via histamine release from the ECL cells (86). Notably, the CCK_2_ receptor is also expressed on human and porcine pancreatic islet cells (87), where it contributes to the gut hormonal incretin effect on insulin and glucagon secretion (88). The CCK_2_ receptor is less specific than the CCK_1_ receptor, as it binds all carboxyamidated CCK and gastrin peptides irrespective of the degree of tyrosyl-sulfation (82, 83).

The differentiated receptor distribution in the gastrointestinal tract illustrates the central hormonal role that blood-borne intestinal CCK peptides play in digestion and metabolism.

#### The plasma measurements

In accordance with the origin of endocrinology, a defining character of hormones has been their traveling via blood to their targets. Consequently, it is in endocrinology necessary to be able to measure the concentrations of hormones in plasma. Both the understanding of basal regulatory and pathophysiological functions of the hormone as well as its impact as biomarker in clinical diagnosis and therapy-monitoring require accurate plasma measurements. And that has been a major challenge for CCK.

Accurate and sophisticated bioassays for plasma CCK were described in the 1980s (70, 89, 90). But the complexity and labor intensiveness precluded, however, wider use in other laboratories. Therefore, the only feasible approach has been development of RIAs with the necessary sensitivity and specificity. It took decades to develop such assays (71, 91, 92, 93). The challenges were first to raise antibodies of very high affinity, because bioactive CCK in plasma from normal subjects circulate in femtomolar to low picomolar concentrations (70, 71, 89, 92). Secondly, the antibodies must in molar terms bind all the different bioactive peptides in circulation (CCK-58, -33, -22, and -8) equally well – in analogy with the CCK_1_ receptor binding; and finally, the antibodies should not bind any of the homologous gastrin peptides. This last criterion has been the largest challenge, because the gastrin concentrations in plasma are about ten-fold higher than those of CCK (70, 71). And in a clinical context, hypergastrinemic patients are not uncommon (94). An additional problem with low-quality and unreliable commercial kits for plasma CCK measurements was recently reviewed (95). In view of the challenges for measurement of the true concentrations of CCK in plasma, users of commercial kits have to evaluate the reliability of each kit carefully.

In summary, since almost all CCK in blood originates from the enteroendocrine I-cells in the gut, specific measurement of CCK in plasma has confirmed that intestinal CCK fulfills all criteria for being a classic blood-borne hormone (see also Fig. 4). But it is not a simple hormone: The molecular heterogeneity and the low concentrations in circulation have to be considered. Moreover, the close structural homology with gastrin as well as their sharing of the CCK_2_ receptor also requires attention in the understanding of CCK as a hormone. And the concept becomes even more challenged with the recognition of expression of CCK also in extraintestinal endocrine cells and non-endocrine cells (*vide infra*).

### Six descriptions of the discoveries of CCK in extraintestinal cells

#### CCK in central and peripheral neurons

It came unexpectedly when Vanderhaeghen *et al.* in late 1975 reported that the vertebrate brain contained a ‘new peptide reacting with gastrin antibodies’ (96). Soon after, three gastrin immunoassay laboratories (from Liverpool, New York, and Aarhus) began to characterize the gastrin-like peptide in extracts of brain tissue (36, 97, 98, 99). In rapid succession, but with the Liverpool-laboratory first, they revealed that the predominant molecular form of the gastrin-like neuropeptide was not a gastrin peptide but sulfated CCK-8 (36, 97, 99, 100). Further studies showed that also longer (CCK-58 and CCK-33) as well as a shorter forms (CCK-5) are expressed in cerebral neurons, although in concentrations lower than those of CCK-8 (36, 60, 80). In accordance with the widespread occurrence of especially the CCK_2_ receptor in cerebral tissues, neuronal CCK peptides turned out to be potent neurotransmitters in all brain regions except the cerebellum (101, 102). For the sake of completeness, it should be noted that true gastrin peptides also are expressed in central and peripheral neurons (36, 103, 104) but considerably more sporadic and in low amounts as compared to the CCK peptides (36, 80).

In fact, the brain in higher mammals expresses more CCK than the gut (Table 1). Moreover, cerebral CCK neurons are more abundant than neurons of any other neuropeptide, giving CCK a unique status as brain peptide (80, 105, 106). While most peptidergic neurons are present in subcortical regions, CCK is expressed in the highest concentrations in neocortical neurons (36, 80, 107). The perikarya of the cortical CCK nerves are distributed in layers II-VI, with the highest frequency in layers II and III (80, 108). CCK in mesencephalic dopamine neurons projecting to the limbic area of the forebrain (105) has aroused some clinical interest because these neurons are supposed to be involved in schizophrenia.

**Table 1:** The expression of cholecystokinin in normal adult mammalian tissue.

Tissue	Tissue concentration^a^ (pmol/g)
Intestinal tract	
Duodenal mucosa	200
Jejunal mucosa	150
Ileal mucosa	20
Colonic mucosa	5
Central nervous system	
Cerebral cortex	400
Hippocampus	350
Hypothalamus	200
Cerebellum	2
Spinal cord	40
Peripheral nervous system	
Vagal nerve	25
Sciatic nerve	15
Nerveplexes in colonic wall	5
Extraintestinal endocrine glands	
Adenohypophysis	25
Neurohypophysis	20
Thyroid gland	2
Adrenal medulla	1
Genital tract	
Testicles	5
Spermatozoas	1
Cardiovascular system	
Atrial myocytes	10
Ventricular myocytes	2
Mononuclear immune cells^b^	++

^a^Orders of magnitude based on measurement of tissue extracts from different mammalian species. ^b^Expression determined only by immunocytochemistry.

Outside the brain, the colon contains numerous CCK neurons, whereas jejunum and ileum are more sporadically innervated (80). Colonic CCK fibers penetrate the circular muscle layer to form a plexus in the submucosa (80). In accordance with these locations, CCK peptides excite colonic smooth muscles and release acetylcholine from neurons in both plexus myentericus and submucosa (109). Ganglionic cell somas and endocrine cells in pancreatic islets are also surrounded by CCK nerves (110, 111). Finally, afferent vagal nerve fibers also contain CCK (112, 113).

The physiologic and pathophysiologic roles of the high concentrations of CCK in the brain are far from settled. But there are indications that cerebral CCK neurons are involved in central satiety regulation and in memory. And clinically, cerebral CCK seems involved in anxiety and – as mentioned – perhaps in schizophrenia (105).

#### CCK in extraintestinal endocrine cells

The CCK gene is expressed also in several well-known endocrine cell types outside the gut. Hence pituitary corticotrophs and melanotrophs express significant amounts of proCCK fragments but the posttranslational processing results in only trace amounts of conventional α-amidated CCK peptides (114, 115). Also, thyroid C-cells produce CCK,but mainly as non-sulfated but amidated CCK-8 (116). Since C-cells also are well equipped with CCK_2_-receptors (117), the thyroid unsulfated CCK-8 is probably an autocrine stimulator of growth of the normal and not least malignant C-cells. Adrenal medullary cells produce small amounts of CCK, although amidated and with a low degree of sulfation (118). The significance of adrenal CCK is so far unknown. Finally, and as mentioned previously, CCK nerve terminals are present also in pancreatic islets, where short molecular forms of CCK can contribute to the regulation of the secretion of islet-cell hormones (111).

#### CCK in male germ cells

It was a major surprise to see that spermatogenic cells – although transiently – express the CCK gene in most mammals (119, 120). Less than 25% of the amidated CCK is sulfated. Interestingly, the CCK peptides in mature spermatozoa are concentrated in the acrosomal granule, which opens the possibility that CCK may play a role in fertilization due to the acrosomal reaction (120). The acrosomal expression is species-specific, as human spermatozoa in addition to CCK also express its homologue, gastrin (121). The reason for the dual expression is unknown.

#### CCK in immune cells

Cholecystokinin immunoreactivity has consistently been found to be expressed in human and rat mononuclear cells in blood (122, 123). Moreover, CCK-8 (sulfated as well as non-sulfated) has been reported to exert a wide spectrum of stimulation and inhibition on lymphocytes, macrophages, and cytokine release, with ensuing anti-inflammatory effects (124, 125, 126, 127). The field is complex due to the many peptide players; but the clinical impact of CCK in inflammatory diseases and endotoxin shock may be significant.

#### CCK in cardiac myocytes

Fetal mice express high levels of CCK mRNA in cardiac myocytes (128). Accordingly, adult cardiomyocytes in mice, rats, and pigs contain substantial amounts of the proCCK protein (129). The processing, however, of cardiac proCCK is unique, as the end product of the posttranslational maturation is a long triple-sulfated and *N*-terminally truncated fragment 25–94 with only trace amounts of the conventionally amidated and sulfated CCK peptides (129). The tissue concentration of the long proCCK fragment is higher in atrial than ventricular myocytes. The proCCK_25-94_ fragment is released to plasma and may find use as a marker of the risk of mortality in heart failure patients (129). The fate of the corresponding N-terminal 1–24 fragment of proCCK has remained obscure in spite of several attempts to find it.

#### CCK in tumor cells

The cholecystokinin gene is expressed at highly variable amounts in different neuroendocrine tumors, especially corticotrophic pituitary tumors (130), medullary thyroid carcinomas (116), phaeochromocytomas (118), and pancreatic islet cell tumors of which some may cause a specific clinical CCKoma syndrome (131, 132, 133, 134). The CCK gene is also expressed in Ewing’s sarcomas, where proCCK is apparently is poorly processed. However, specific proCCK measurements may be used to monitor the treatment of sarcomas (135). Cerebral gliomas, astrocytomas, and acoustic neuromas also express CCK peptides (136, 137, 138). The present knowledge about tumor expression of CCK has been summarized in a recent review that also discusses measurements of CCK and proCCK in plasma as tumor markers (139).

## Concluding comments

Since the structural identification of CCK half a century ago as a single peptide with a sequence of 33 amino acid residues (CCK-33), the CCK story has been full of major, unexpected revelations: First, it was shown that the bioactive C-terminus of CCK was similar to that of the gastrins and those of amphibian skin peptides as well as protochordean and insect neuropeptides. Moreover, CCK and gastrin peptides all turned out to be agonists for one of the two CCK receptors, the CCK_2_ receptor. In the late 1970s and in the 80s, it was also demonstrated that bioactive CCK occurs in multiple molecular forms – from CCK-58 to CCK-5 with and without tyrosyl O-sulfations – as a consequence of complex, cell-specific posttranslational maturation processes. At variable intervals, it was subsequently shown that CCK peptides are expressed all over the body: abundantly in central and peripheral neurons as potent neurotransmitters, in intestinal and extraintestinal endocrine cells as classical hormones, in germ cells as putative fertility factors, in cardiac myocytes for unknown reasons, and in immune cells of significance for inflammatory diseases. Finally, the proCCK maturation appears to be cell specific also in tumors expressing the CCK gene. The tumors therefore release a multifaceted pattern of CCK peptides that may cause a specific CCKoma syndrome. In summary, CCK should today be seen as a rather ubiquitous system of intercellular messenger peptides.

A point is, however, that CCK is only one example of a hormonal peptide system with a molecular and cellular complexity as described previously. In fact, all gastrointestinal peptide hormones (ghrelin, gastrin, secretin, the gut glucagons, neurotensin, the tachykinins, somatostatin, etc.) are also complex systems widely expressed in multiple bioactive forms both within and outside the gastrointestinal tract. And similar features are seen for extraintestinal peptide hormones such as the calcitonins, parathyroid hormones, and neuropeptides originally discovered in extracts of central and peripheral nervous tissue. In other words, most peptide hormones have a wide range of activities, of which only some are due to peptide messengers traveling via blood (for reviews, see 140).

This situation challenges the classical hormone concept. Etymologically, the Greek origin of the word ‘hormone’ (hormoa) means ‘I arouse to activity’, which is exactly what bioactive peptides do irrespective of the routes to their targets. Consequently, there are now biological as well as etymological reasons for expansion of the hormone concept to cover all bioactive messenger molecules whose target cells express specific receptors.

Ernest Starling was in good faith when he introduced the word ‘hormone’ as a designation for blood-borne messenger molecules 116 years ago (1). Secretin and blood-borne regulation as an alternative supplement to neural regulation was indeed a decisive paradigmatic shift in physiology (1, 2). Starling could not know that the same peptides acted both as neurotransmitters and blood-borne messengers – not to speak of further roles as growth factors, fertility factors, cytokines, myokines, and adipokines.

## Declaration of interest

The author declares that there is no conflict of interest that could be perceived as prejudicing the impartiality of this review.

## Funding

This work did not receive any specific grant from any funding agency in the public, commercial or not-for-profit sector.
